# Is early treatment of disseminated intravascular coagulation beneficial in septic patients?

**DOI:** 10.1186/cc13971

**Published:** 2014-07-04

**Authors:** Hideo Wada, Takeshi Matsumoto, Yoshiki Yamashita, Tsuyoshi Hatada

**Affiliations:** 1 Department of Molecular and Laboratory Medicine, Mie University Graduate School of Medicine, Tsu, Mie 514-8507, Japan; 2 Blood Transfusion Center, Mie University Hospital, Tsu, Mie 514-8507, Japan; 3 Department of Hematology and Oncology, Mie University School of Medicine, Tsu, Mie 514-8507, Japan; 4 Emergency Critical Care Center, Mie University Hospital, Tsu, Mie 514-8507, Japan

## 

We read with interest the recent issue of *Critical Care*, particularly the article by Gando and colleagues [1] about the validation of the scoring systems for disseminated intravascular coagulation (DIC). Mortality in patients with DIC according to diagnostic criteria of the Japanese Association of Acute Medicine (JAAM; 31.8%) was similar to that in patients with International Society of Thrombosis and Haemostasis (ISTH) overt-DIC (30.1%). A previous report [2] showed different results; mortality was significantly higher in patients with overt-DIC (34.4%) than in those with JAAM DIC (17.2%). The difference in the mortality between this report [1] and the previous report [2] may depend on not only the sensitivity of the diagnostic criteria, but also on the antithrombotic therapy (ATT).

Although most patients were considered to be treated with ATT at the early stage of DIC in this study [1], those treated in the other study [2] had late stage DIC [3]. As the presence of neutrophil extracellular traps [4] and hypercoagulation in DIC induce localization of infection, the administration of ATT may spread the infection. Therefore, ATT may worsen sepsis in the early stage of the disease while improving hemostatic abnormalities following organ failure in patients with severe sepsis. Overall, ATT may not improve the outcomes of patients with sepsis in the early stage, although it can potentially improve the outcomes of those with overt-DIC (Figure 1). The timing of ATT may be too early in septic patients when using the JAAM diagnostic criteria and too late in those with ISTH overt-DIC.

**Figure 1:**
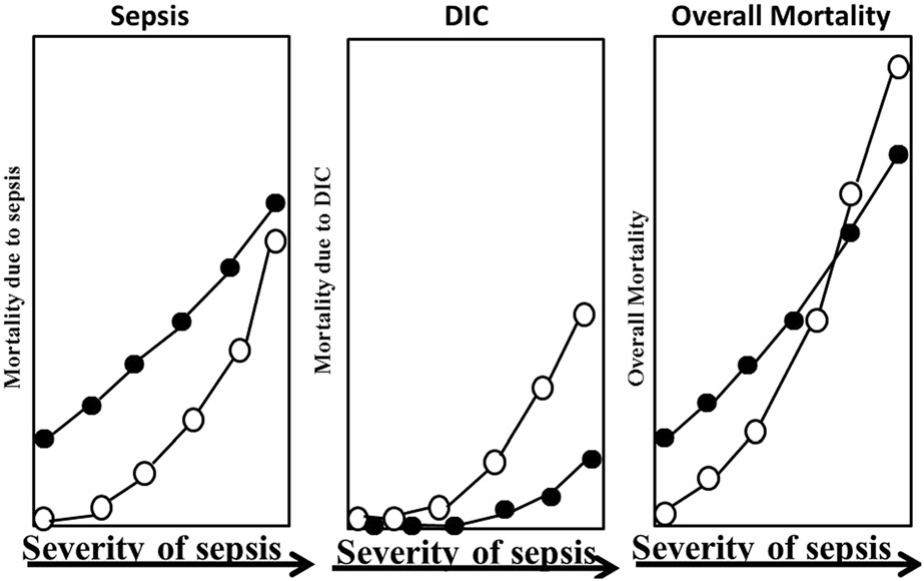
**Mortality due to sepsis and/or disseminated intravascular coagulation (DIC).** Open circles indicate without DIC treatment, closed circles with DIC treatment.

## Authors’ response

Satoshi Gando

We appreciate the interest of Wada and colleagues in our recently published article studying the JAAM DIC scoring system in patients with severe sepsis [1]. It is now widely accepted that localized platelet and fibrin thrombosis at the site of infection is a physiologic reaction that protects against dissemination of microorganisms and pathogen-associated molecular patterns derived from them into the systemic circulation, which is now called immunothrombosis [5]. Therefore, we agree that early anticoagulation therapy for sepsis probably induces uncontrolled immunothrombosis, leading to pathological systemic DIC. Fourrier [6] clearly demonstrated that the target for the treatment of sepsis is not sepsis itself, but DIC as a result of the overwhelming effects of sepsis on immunothrombosis. The mortality rate of the severe sepsis patients who met the JAAM DIC criteria was 31.8%, and the Kaplan-Meier curves clearly demonstrated that there was a lower 1-year survival rate in the JAAM DIC patients, which supports our opinion that the DIC patients diagnosed by the JAAM scoring system should be treated as early as possible [1]. The differences in the mortality rate between the two studies pointed out by Wada and colleagues were due to differences in the subjects included in the two studies [1,2]. Our previous study included diverse clinical conditions that were associated with DIC [2], but the inclusion criteria for the current study were restricted to only severe sepsis patients [1].

## Abbreviations

ATT: antithrombotic therapy; DIC: disseminated intravascular coagulation; ISTH: International Society of Thrombosis and Haemostasis; JAAM: Japanese Association for Acute Medicine.

## Competing interests

The authors declare that they have no competing interests.
